# Postpartum consultation: Occurrence, requirements and expectations

**DOI:** 10.1186/1471-2393-8-29

**Published:** 2008-07-23

**Authors:** Ingrid Carlgren, Marie Berg

**Affiliations:** 1 Department of Obstetrics, Sahlgrenska University Hospital, SE 416 85 Gothenburg, Sweden; 2 Sahlgrenska Academy, Institute of Health and Care Sciences, University of Gothenburg, Box 457, SE 405 30 Gothenburg, Sweden

## Abstract

**Background:**

As a matter of routine, midwives in Sweden have spoken with women about their experiences of labour in a so-called 'postpartum consultation'. However, the possibility of offering women this kind of consultation today is reduced due to shortage of both time and resources. The aim of this study was to explore the occurrence, women's requirements of, and experiences of a postpartum consultation, and to identify expectations from women who wanted but did not have a consultation with the midwife assisting during labour.

**Methods:**

All Swedish speaking women who gave birth to a live born child at a University Hospital in western Sweden were consecutively included for a phone interview over a three-week period. An additional phone interview was conducted with the women who did not have a postpartum consultation, but who wanted to talk with the midwife assisting during labour. Data from the interviews were analysed using qualitative content analysis.

**Results:**

Of the 150 interviewed women, 56% (n = 84) had a postpartum consultation of which 61.9% (n = 52) had this with the midwife assisting during labour. Twenty of the 28 women who did not have a consultation with anyone still desired to talk with the midwife assisting during labour. Of these, 19 were interviewed. The content the women wanted to talk about was summarized in four categories: to understand the course of events during labour; to put into words, feelings about undignified management; to describe own behaviour and feelings, and to describe own fear.

**Conclusion:**

The survey shows that the frequency of postpartum consultation is decreasing, that the majority of women who give birth today still require it, but only about half of them receive it. It is crucial to develop a plan for these consultations that meets both the women's needs and the organization within current maternity care.

## Background

One important aim of care during childbirth is a positive birth experience for the woman. Several issues have an impact on this such as; expectations, age, civil status, unemployment and previous negative birth experience [1-3]. Individual women have varying capacities to handle challenges, disappointments and sorrows in connection with childbirth, and their abilities to handle their demands on themselves realistically also vary. If the experience of labour does not respond to expectations, senses of meaninglessness and of not mastering the situation are frequent among mothers [1]. Long-term studies of women's memories of childbirth have showed that 15–20 years afterwards, they remember vividly the words and actions of caregivers [4]. Supportive care may protect some women from a long-lasting negative experience [5]. Of special value for a positive experience is the first encounter between the woman giving birth and the midwife [6-10]. A positive encounter includes seeing and listening to the woman, trying to understand her and meeting her needs and experiences. This type of respectful meeting gives the woman a feeling of security during labour and strengthens her self-confidence [7-9,11]. Another central issue is the midwife's ability to be present during labour [7,8,11].

In Sweden the care is shared between two health care organizations; primary health care during pregnancy, and hospital care during childbirth and the first post-natal week. Midwives are the primary care-givers and responsible for normal pregnancy and childbirth. In case of high obstetric risk they still work very independently, however, under supervision of obstetricians [12]. Antenatal care consists of regular appointments with the aim of detecting symptomless complications and giving psychosocial support and health education. There is also a follow-up provided with the midwife at the antenatal care unit about two months after childbirth. Since the late 70s, Swedish midwives assisting during childbirth, have performed a so-called 'postpartum consultation' with women about their experiences. However stringent routines are lacking; both content and time for the consultation vary. Mostly the consultation are performed without an appointed time, merely when the midwife has time available. Some consultations are carried out immediately after childbirth, some on the postnatal ward and some via telephone contact to the home. In general, midwives have no special education for this part of their duties.

In modern streamlined maternity care, including shortened postpartum care time and fewer resources, the possibility of offering women a postpartum consultation is reduced. A population-based Swedish study from the 90s shows that 80% of the women had a postpartum consultation either with a midwife or an obstetrician from the delivery ward [13]. Another Swedish study from the late 90s shows that 66% of the primipara women and 74% of the multipara women wanted a postpartum consultation [14]. An Australian randomized study using postpartum debriefing, a special form of postpartum consultation, shows that 94% of the women were positive about this debriefing [15]. More knowledge is needed to attain evidence-based care routine. The aim of this study was therefore to explore occurrence, and women's requirements and experiences of the postpartum consultation, and to identify expectations from women who wanted but did not have a consultation with the midwife assisting during labour.

## Methods

### Settings and study population

In order to study the occurrence of postpartum consultation a retrospective study was conducted at one of the three delivery wards at Sahlgrenska University Hospital in Gothenburg, providing obstetric care to approximately 3500 women per year. The midwives worked both on the delivery ward and on the postnatal care ward. The aim was that at least 80% of the women should be offered a postpartum consultation either by the midwife assisting during childbirth or a midwife on the postnatal care ward. Some midwives offered a consultation to all women, some only to those with complications such as vacuum extraction or emergency caesarean section. The consultations were usually carried out before the women went home. The duration of the consultation varied in accordance to the woman's need or to the midwife's work load. Usually no specific time for the consultations was appointed; the midwife visited the woman when she had time and this was usually at the end of the working day. If the postpartum stay at the hospital was less than 72 hours the family came back for another check-up of the baby, which included a possibility to have a short talk about experiences of childbirth and postpartum care. Postpartum consultation could also be performed by the midwife at the antenatal care unit. Most midwives had no special education in how to conduct a postpartum consultation; some were trained in debriefing consultation.

All Swedish speaking women with live born children were consecutively included over a three-week period (n = 175). The participation was on a voluntary basis. If the woman during the interview indicated further need for talking with a professional or wanted to meet the assisting midwife, this was taken into consideration and was arranged by the interviewer. Ethical consideration was in accordance with the Swedish Ethical Appeals law. Approval for the study was obtained from the head of the participating hospital obstetric department.

### Procedure

Data collection was carried out in two steps; one telephone interview with all women, and a second one with women who did not have a consultation but had wanted to have one with the midwife assisting during childbirth. The first structured phone interview was performed 6 – 8 weeks after childbirth by two midwives who had not assisted the women during childbirth. Information regarding the aim of the study was given and complete confidentiality was assured. The questions at issue are described in Table 1. The second telephone interview was performed 6 – 8 months after childbirth. After consenting to participate, the following questions were asked: What did you wish to talk about with the midwife assisting during labour? Do you still wish to talk to the midwife assisting during labour? During the interview notes were taken by the interviewer. To assure that the answers were understood in a correct way the notes were read to the woman who could affirm or correct the notes. The notes were transcribed immediately after each interview.

**Table 1:** Questions at issue during the first phone interview

• Did the woman have a talk about her experience of labour with some staff category within the health care sector through a personal meeting or by phone?	
• With whom did the woman have a talk about her experience of labour?	
• Did the woman wish to have a talk about her experience of labour but didn't get one?	
• Was it important for the woman to have a talk about her experience of labour?	
• Is the woman satisfied with the talk she had about her experience of labour?	

### Data analysis

The data from the first structured phone interviews were compiled according to frequency and median value. The text from the second phone interviews was analysed by qualitative content analysis. It was read several times, divided into meaning units, and to codes which were subsequently consolidated into subcategories and categories [16]. Examples of the analysis process are described in Table 2.

**Table 2:** Example from the analysis process from the second phone interview

**Meaning units**	**Condensed ** **Meaning units**	**Code**	**Subcategory**	**Category**
Wanted to review the labour with the midwife assisting	Review the labour process	Course of labour	To have events and experiences explained	
One is not hundred percent aware	Not hundred percent aware	Memory gaps	To have memory gaps from labour filled in	To understand the course of events during labour
I wanted to get her view of labour, if everything was like it was supposed to be	Was everything like it was supposed to be	Normality	To know if the labour process was normal and if they had acted in the correct way	

It was so confusing when the body wanted to go on pushing and the midwife said that it was not yet time	Confusing when the body wanted to go on pushing, the midwife said it was not yet time	Trust one's body or the midwife?	Hovering between trust in own body and trust in the midwife	To describe own behaviour and feelings

## Results

A total of 150 women out of 175 were interviewed in the first phone interview. The women were in the age range of 18 – 46 with a median value of 31 years, 76 were primipara, 54 two-para and 20 multipara. Four women were single. A total of 56% (n = 84) had the postpartum consultation, 36 women had it on two or more occasions, either with the midwife or the obstetrician at the postpartum care unit or with the midwife in the public health care system. A few had the consultation with the nurse at the post natal well-child clinic. A total of 121 consultations were conducted. Fifty-two women (61.9%) had a consultation with the midwife assisting at childbirth. A total of 69 women were satisfied with the consultation and seven did not have any opinion. Eight women (10%) were not satisfied with the consultation either because the consultation came too early, because it did not include talk about their experience of labour, because the time period was too short or because another person was present.

**Figure 1:**
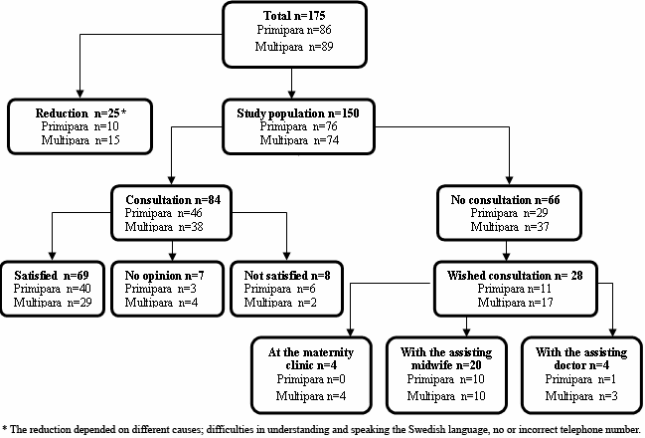
Schematic view over the study population and the results from the first phone interview.

Slightly less than half of the women (n = 28) who did not have the consultation stated that they did need one. Twenty of these wanted to have a talk with the midwife assisting during labour. Nineteen of them; ten primipara and nine multipara, had a second telephone interview six to eight months after labour (Figure 1). They were in the age range of 25 – 42 years with a median value of 31 years, all were married or cohabited with a partner, one woman was of foreign origin. One of the women had an elective caesarean section, one had a vacuum extraction and seventeen women had normal vaginal labour of which seven had a very fast delivery. Only one of the women remained with a need for a consultation with the midwife assisting during labour. The reason for no longer wanting a consultation was that too much time had passed and the respondent thought that the midwife probably would not remember the course of labour, but also that the experience had been worked through together with the respondent's husband or friends or by writing down the experience. In addition, the phone interview itself, which offered the respondents the opportunity to ventilate feelings, was beneficial and resulted in no need for a consultation for some women.

The reasons for women's need to talk were summarized in four categories with subcategories validated by quotations (Table 3). The women primarily wanted to talk about the experience of labour but also about the time after, about postpartum care and the future. The four categories are described below, the characters ' ' denotes subcategories, quotations are in italics, the character // denotes that quotations are from several different women.

**Table 3:** Issues the woman wanted to talk about in a postpartum consultation

**Category**	**Subcategory**
To understand the course of events during labour	To know if the labour process was normal and if they had acted in the correct way
	To have events and experiences explained
	To have memory gaps from labour filled in
	To reconstruct a fast course of labour

To put into words, feelings about undignified management	Feeling of being betrayed
	The lack of feedback
	The lack of information
	To not be trusted

To describe own behaviour and feelings	To put into words own effort
	Losing one's footing
	Hovering between trust in own body and trust
	in the midwife

To describe own fear	Fear of labour
	Fear of damage to own body
	Fear for the health of the child

### To understand the course of events during labour

The women wanted 'to know if the labour process was normal and if they had acted in the correct way'. If this information was lacking a feeling of uncertainty was present. They did not dare trust own feelings when they did not know what was considered as a normal labour process.

*Everything went very well, only I didn't know it at the time. I wanted in some way to be confirmed...that everything was as it was supposed to be and that I did it correctly //Now, after listening to the other parents in the group I understand that I had an easy delivery, but I wasn't aware of that at the time. I probably wanted to get that confirmed*.

The experience of labour was described simultaneously as dramatic, shocking and positive. However, to be able to understand the experience in total, there was a need 'to have events and experiences explained'. Dramatic experiences were for example premature labour, labour with a vacuum extraction, episodes with foetal distress or the experience that "the child was stuck." There were also concerns about the analgesia given, if it really was necessary and how things would be next time in labour. Some women had their own explanations for the labour process which they needed to have confirmed. One woman thought that her fear of labour was the reason for premature birth. Another woman wondered what had happened with her body since it was so painful. One woman expressed her feelings in the following way:

The heart sound of the child dropped and stayed low for a long time. The midwife became worried and everything just became confusing. I had just received analgesia and I wanted to know more. Why did this happen? Was it because I had an epidural?

Memory gaps were described as something very unpleasant. Women were aware that the gaps were because they had not been totally present or conscious during the intensive labour pains. In order to understand what they had experienced and to get a clearer picture about the process, they needed 'to have memory gaps from labour filled in'. In a fast course of labour the experience of not 'keeping up' was difficult. Often the perspective of time was missing and the women had questions like: What was happening? Why was it so painful? Then there was a wish 'to reconstruct a fast course of labour' together with the midwife, to go through the course of labour step by step, including cervix dilatation and the point of time when pain relief was given. In addition, there was a wish for an explanation of why it happened so fast.

*After she put the (intravenous) drip I can't remember what happened. I only remember the last phase. It was horrible with all the memory gaps.// If you give birth too fast your body doesn't recognize what's happening // It would've been nice to talk about it, there are so many thoughts and feelings but it wasn't strenuous, rather a positive experience. Everything happened so fast, in the end I was confused, I wish I could have...that all the pieces fell into place*.

### To put into words, feelings about undignified management

Central in almost all the women's descriptions were the feelings of being treated in a disrespectful way during labour or postpartum care. This gave a 'feeling of being betrayed', and occurred for example when the midwife did not pay attention to woman's written wishes for labour pain relief, or when the midwife did not come to a promised postpartum consultation. Some women expressed feeling lonely and insecure during the period of the postpartum stay as they had to take care of themselves. Others, especially multipara women, felt that the midwives did not care about them since everything had been normal.

*I felt very lonely this time. I have given birth twice before and I thought it was great...Now, nobody asked me how I felt and nobody care. I really felt lonely*.

'The lack of feedback' also gave a feeling of being met in a disrespectful way. This concerned thoughts about the next labour, comparison with earlier labours, or simply the opportunity to thank the midwife. Unsatisfied women wanted to put words on this in order to be able to let go of negative thoughts. These thoughts concerned both 'hard things' such as broken equipment, or pure dissatisfaction about the professional management of labour. Another reason for talking was that they felt a responsibility to tell what happened in order to prevent other women from having such a negative experience.

*I have been thinking about it a lot...that I didn't get to talk to her. It felt worse in the beginning but it still hasn't disappeared...not that I feel bad about it but I don't want to look them in the face again. I feel disappointed and deceived in some way. In order to let go, I would really have needed to talk to her*.

Another essential issue of disrespectful management was 'the lack of information'. Continuous information was considered to eliminate anxiety and fear and to increase a woman's own strength and feeling of safety and participation. Lack of information could, for example, concern how the woman's labour was proceeding, if everything was normal, how much time was left before the child was born or what was considered as normal bleeding after labour. Some women missed information about perineal ruptures; how long it would take to be completely healed, how many sutures, if the sutures were supposed to be removed or where the sutures were put. Lack of information about the labour process could reduce a woman's strength:

*I wanted more information, my husband didn't know if he was allowed to say anything, that he saw a glimpse of the head. If I had known that, then maybe I would've had more unimaginable strength*.

Another disrespectful behaviour that needed to be put into words was 'to not be trusted'. This could, for example, arise in an acute situation when the women felt totally worn out and exposed and the staff said: "*pull your self together*." One woman got this feeling when the midwife did not allow her to bear down even though she felt she should. Another one felt this when the midwife misjudged a fast labour process, and yet another when the midwife estimated that the labour had not yet started and sent the woman home:

*I knew that I was going to give birth but the midwife wanted to send me home, then, two and a half hour later, I gave birth*.

### To describe own behaviour and feelings

For the women it was also essential 'to put into words their own effort' during labour. Feelings varied and could be both difficult and pleasant, such as helplessness, failure, shame and pride. One woman with a premature labour felt like failure; she blamed herself for going into premature labour. Another woman was ashamed for being whiny and unpleasant to the midwife. She dwelt on this and had a need to decrease her burden of shame with a postpartum consultation by apologizing and explaining herself. To put words on feelings of pride could, for example, include giving birth to a beautiful child, when succeeding with vaginal birth after an earlier fear of childbirth, or after a prior caesarean section, or as the following woman stated:

*Labour progressed and I only used laughing gas, I'm proud of that*.

Another feeling to put into words was the feeling of 'losing one's footing' which could happen in an unpleasant or unmanageable situation, such as when blood pressure drops after an epidural or when labour was experienced as one long labour pain:

*I can't remember, I almost lost my footing, right when I thought the pain was about to decrease another one came*.

It was also essential to describe feelings of 'hovering between trust in own body and trust in the midwife'. Many times the trust in the midwife's judgment took over the trust of own body of which the following quote is an example:

*It was so confusing when my body wanted to push but the midwife told me that it was not time yet*.

### To describe own fear

Another aspect some women needed to talk about was their 'fear of labour' often connected with distrust in themselves and their capability to give birth.

*I panicked; I was very scared of giving birth, afraid to not be able to handle it, of not being prepared to give birth. I was in despair, thought I wouldn't be able to handle it*.

Women also felt 'fear of damage to own body'. This pertained to worries for rupturing at labour, fear of negative effects of analgesics like an epidural, or as described in the following quote:

*I would've liked to talk about when they inserted that catheter. I wanted to be under a general anaesthesia...I was frightened, so afraid that it would hurt. I had no idea...I could've found out before...got some more information, that it didn't hurt, then I wouldn't have been so frightened, because it didn't hurt. I could've found out the day before*.

Another obvious feeling for the women was 'fear for the health of the child'. Even if everything had gone well an anxiety could persist over what could have happened. A woman with a premature labour feared that it had caused brain damage in her baby. She had not been able to talk about it with anyone, the anxiety was constantly present and she searched for abnormalities. Another woman felt that the midwife's worries had been transferred to herself:

*The heart sound of the child dropped... The midwife became worried and everything just became confusing...I probably didn't really understand what was happening. It was only afterwards that we found out what could've happened. I think I felt quite secure after everything calmed down, but now afterwards we've found out that the child could've died*.

## Discussion

For future well-being and possible pregnancy/childbirth, it is of outmost importance that the woman has adapted to her childbirth experience. The post partum discussion may be an important instrument in this process. The survey shows that the occurrence of postpartum consultation has decreased in comparison to the results from studies in the 90s [13]. Possible reasons for this may be reduced work force with less time and fewer resources, as well as a lack of research-based knowledge of women's needs. Our result show that the need for the postpartum consultation remains high since 74% of the women considered it to be important. This is verified by other studies that show the importance of offering women postpartum consultation, preferably with the midwife assisting during labour [14,17-19]. Many women had consultations with different staff categories, even late during the postpartum phase. In accordance with other research, this study indicates that women have a need for a follow-up consultation during the postpartum period [14,20-22]. According to earlier research, consultation by phone may be a good alternative form of follow up in modern care which is signified by short care time. This is a common and popular, non threatening form of contact [23]. There are similarities between conducting a postpartum consultation by phone and conducting a phone interview like the ones in this study. Through the open nature of the questions a mutual relationship was developed which paved the way for women's understanding of their experiences and eliminated their need of further consultation.

Our result show that a primary need for the women was to have an explanation and an understanding for what had happened during labour, regardless of 'normal' or 'abnormal' episodes. This reflects what has been described in the salutogenetic theory; that an experience must be comprehended before it is manageable. Thereafter a feeling of meaningfulness can occur, and a sense of coherence may be obtained [24]. For a mother to a new-born a sense of coherence increases well-being and self-esteem which facilitates the transition to motherhood. Essential to understanding an episode in its entirety is to find all "the pieces of the puzzle" and put them together. The interviewed women had lost pieces, i.e. had memory gaps and had experiences brought on by exhaustion, medication or storming emotions which needed to be explained, as described in other research [25]. Due to fast course of labour, seven of the women had negative or incomprehensible experiences. We can speculate that the risk for this group of women of not obtaining a postpartum consultation is probably higher, as they often had 'a normal childbirth' in medical terms.

Not being treated in a respectful way was expressed as insulting by the affected women, who needed to put their experience into words. One reason for this negative feeling was a lack of information. The importance of information as part of care is a well-known and often mentioned in policy documents, such as laws for health- and medical care [7-9,18]. Mothers to new-borns have a constant need for information, and the challenge is to inform the individual woman or family sufficiently, but not too much as this may cause confusion, insecurity and loss of self-esteem [26].

The interviewed women had a need to describe own behaviour and feelings. This included positive feelings such as a sense of pride over giving birth to a child despite fear, without or with minimal pain relief. Mainly, the women needed to put into words negative feelings such as helplessness, failure and shame. When labour became too overwhelming and a lack of control such as "losing one's footing," was experienced, feelings of distrust of their own body and their own capacity were present. Lack of control has, in other studies, been found to be an important risk factor for a negative labour experience [2]. Negative feelings and depressed moods are common after childbirth. For health professionals, and especially for midwives in postpartum consultations, it is important to identify these negative feelings and to convince the woman that she had acted sufficiently according to her own capacity during child birth. It is also important to pay attention to symptoms of posttraumatic stress syndrome.

Another essential need the interviewed women revealed was to talk about their fears in connection with childbirth. This could be fear during labour, fear that their body would be damaged during labour and fear for the child's health. Fear as a phenomenon has become common in the population, especially in connection with childbirth. Perhaps this is a consequence of modern life where striving for control and security is essential. Childbearing is connected with the unknown, and increases the need to let go and just 'go with the flow' which for many women is difficult and may lead to fear. Health care professionals, not the least midwives, have to take signs of persistent fear after childbirth seriously as this is a common reason why multiparous women not daring to try another vaginal childbirth or to get pregnant. Research has shown that women with a fear of labour, with a prior planned caesarean section, or with previous negative birth experience have increased risks for a negative experience during future childbirth [2,27]. A postpartum consultation is therefore an important tool to identify women who need professional support to work with their fear. If it is the midwife assisting during childbirth or another midwife with access to medical records who should conduct this consultation may depend on with whom the woman has developed a relationship. Our study shows that the majority of the women who did not have the postpartum consultation but stated that they did need one, wished to have the consultation with the midwife who had assisted at birth. This is an important question for further study.

One limitation of the study is that the women were not prepared for the telephone interview. This meant that some women were disturbed by a crying baby or siblings who wanted her attention. Furthermore, it was not technically possible to tape-record the interviews. However, all written interviews were read up aloud for and confirmed by each woman.

## Conclusion

The survey shows that the frequency of postpartum consultation is decreasing, that the majority of women who give birth today still require it, but only about half of them receive it. It is crucial to develop a plan for these consultations that both meets the women's needs and the organization within current maternity care. Further studies, such as a RCT with different types of interventions, are required to be able to develop good evidence-based postpartum consultation.

## Competing interests

The authors declare that they have no competing interests.

## Authors' contributions

IC carried out the design of the study, read the literature, did the interviews, analysed the interviews and mainly wrote the manuscript. MB assisted with interpreting the result and writing the manuscript. Both authors read and approved the final manuscript.

## Pre-publication history

The pre-publication history for this paper can be accessed here:


